# Sub-cycle dynamics in relativistic nanoplasma acceleration

**DOI:** 10.1038/s41598-019-43635-3

**Published:** 2019-05-13

**Authors:** D. E. Cardenas, T. M. Ostermayr, L. Di Lucchio, L. Hofmann, M. F. Kling, P. Gibbon, J. Schreiber, L. Veisz

**Affiliations:** 1 Max-Planck-Institut für Quantenoptik, Hans-Kopfermann Strasse 1, 85748 Garching, Germany; 2 Ludwig-Maximilian-Universität München, Am Couloumbwall 1, 85748 Garching, Germany; 3 Forschungszentrum Jülich GmbH, Institute for Advanced Simulation, Jülich Supercomputing Centre, D-52425 Jülich, Germany; 4 Centre for Mathematical Plasma Astrophysics, Department of Mathematics, KU Leuven, Celestijnenlaan 200B, 3001 Heverlee, Belgium; 5 Department of Physics, Umeå University, SE-901 87 Umeå, Sweden

**Keywords:** Nanophotonics and plasmonics, Plasma-based accelerators, Ultrafast photonics, Laser-produced plasmas

## Abstract

The interaction of light with nanometer-sized solids provides the means of focusing optical radiation to sub-wavelength spatial scales with associated electric field enhancements offering new opportunities for multifaceted applications. We utilize collective effects in nanoplasmas with sub-two-cycle light pulses of extreme intensity to extend the waveform-dependent electron acceleration regime into the relativistic realm, by using 10^6^ times higher intensity than previous works to date. Through irradiation of nanometric tungsten needles, we obtain multi-MeV energy electron bunches, whose energy and direction can be steered by the combined effect of the induced near-field and the laser field. We identified a two-step mechanism for the electron acceleration: (i) ejection within a sub-half-optical-cycle into the near-field from the target at >TVm^−1^ acceleration fields, and (ii) subsequent acceleration in vacuum by the intense laser field. Our observations raise the prospect of isolating and controlling relativistic attosecond electron bunches, and pave the way for next generation electron and photon sources.

## Introduction

The collective response of electrons in a nanomaterial to intense few-cycle (<5 fs) laser pulses^1^ enables unprecedented spatio-temporal control over electron dynamics^2,3^ and electron emission^4^. Sub-cycle control becomes feasible by manipulating the waveform of the incident field, e.g. by changing the carrier-envelope phase (CEP)^5^. At laser intensities below the damage threshold of the nanomaterial, the nanoscale localization of electromagnetic fields has become a versatile tool for fundamental research^6^ as well as applications^7,8^, including nanoscale electron accelerators, where the accelerating near-field reaches a few-GVm^−1^ resulting in electron kinetic energies in the keV level^9–12^ for intensities below 10^14^ Wcm^−2^. When driven by a waveform-controlled few-cycle laser^13^, the near-field acceleration can lead to isolated electron bunches^14^. While the incident laser radiation is characterized by its maximum amplitude ${E}_{L,0}$, angular frequency ${\omega }_{L}$ or wavelength ${\lambda }_{L}$ and CEP ${\phi }_{CEP}$, the evanescent near-field can be characterized by a decay length ${l}_{d}$ describing its exponential fall-off away from the surface – see Supplementary Materials (SM). For wavelength-sized particles, the scattering of light can be understood by the Mie theory, where the near-field distribution for spherical and cylindrical nanostructures is size-dependent, and its maximum amplitude follows the classical Mie angular dependence. These angles are about 90° off-axis for small targets (≪*λ*_*L*_) in the dipole regime, and tilt towards the laser propagation direction for wavelength-sized objects^4^. The motion of a free electron born under these conditions in a linearly polarized laser field is described by a “quivering” amplitude of ${l}_{q}=e{E}_{L,0}/({m}_{e}{\omega }_{L}^{2})$ and an average energy corresponding to the ponderomotive energy ${U}_{p}={m}_{e}{c}^{2}\sqrt{1+{a}_{0}^{2}/2}-1$, where *e* and *m*_*e*_ are the electron charge and mass, *c* is the speed of light in vacuum and ${a}_{0}=|e{E}_{L,0}/({m}_{e}{\omega }_{L}c)|={[{I}_{L}({{\rm{W}}{\rm{c}}{\rm{m}}}^{-2}){\lambda }_{L}^{2}({\mu {\rm{m}}}^{2})/1.37\times {10}^{18}]}^{1/2}$ is the normalized vector potential of the laser with intensity *I*_*L*_. Recently, sub-cycle electron emission has been reported^6,15^ in the low intensity regime (*a*_0_ ≪ 1), when the emitted electron leaves the accelerating near-field within a half oscillation, i.e. *l*_*d*_/*l*_*q*_ ≪ 1. Here, the traditional quivering picture breaks down and the energy scaling differs substantially from the ponderomotive prediction.

On the other hand, highly intense pulses (*a*_0_ > 1, i.e. *I*_*L*_ > 10^18^ Wcm^−2^) from TW-PW lasers generate MeV-GeV femtosecond electron bunches^16–20^ in gas targets or keV-MeV electrons^21–24^ from solid or overdense plasma targets. Furthermore, direct vacuum laser acceleration (VLA)^25–30^ utilizing these lasers has also been proposed due to its accelerating field, >1 TVm^−1^, significantly exceeding that of low-density plasma accelerators (≈0.1 TVm^−1^). Only the sub-cycle confinement of these relativistic interactions^31^, where the electron oscillatory velocities are comparable to the speed of light, would generate sub-fs electron pulses^32,33^, providing even isolated bunches with quasi-single-cycle lasers^34,35^. However, energy limitation of the necessary few-cycle laser technology has so far restricted the attosecond control to non-relativistic interactions^36^. Nanostructure-assisted interaction^37–39^ and electron acceleration^40,41^ has been hardly accessible to high-intensity TW and not to PW lasers due to their long duration (>20 fs) and poor high-dynamic-range temporal contrast in the ps to ns range that modifies or even destroys the target before the peak of the pulse. Former experiments generating relativistic electrons^40,41^ utilized surface plasmon (SP) polaritons on gratings and thus prohibiting the generation of electron bunches in the attosecond regime. On the other hand, predictions for localized SPs^1^ in nanoplasmas are very promising^35^ for *a*_0_ ≫ 1 including novel features such as intensity dominance over the final electron directionality, appearance of attosecond MeV electron bunches, or enlargement of the skin depth $({\delta }_{p}^{\ast })$. Since in relativistic plasmas ${l}_{d}/{l}_{q}\propto {a}_{0}^{-3/2}$, the sub-cycle emission is expected at a larger extent (see SM). In this work, we gain new insight on the sub-cycle nature as well as electric field dependence on the nanoscale electron acceleration. The experimental findings are supported by 3D particle-in-cell (PIC) simulations. We find a two-step mechanism, where high-charge electron pulses are emitted and pre-accelerated in the sub-cycle near-field of a sub-wavelength-sized plasma formed originally from a tungsten nanotip, and subsequently further accelerated in vacuum by the laser field.

## Results

### Relativistic nanoplasmas

**Figure 1:**
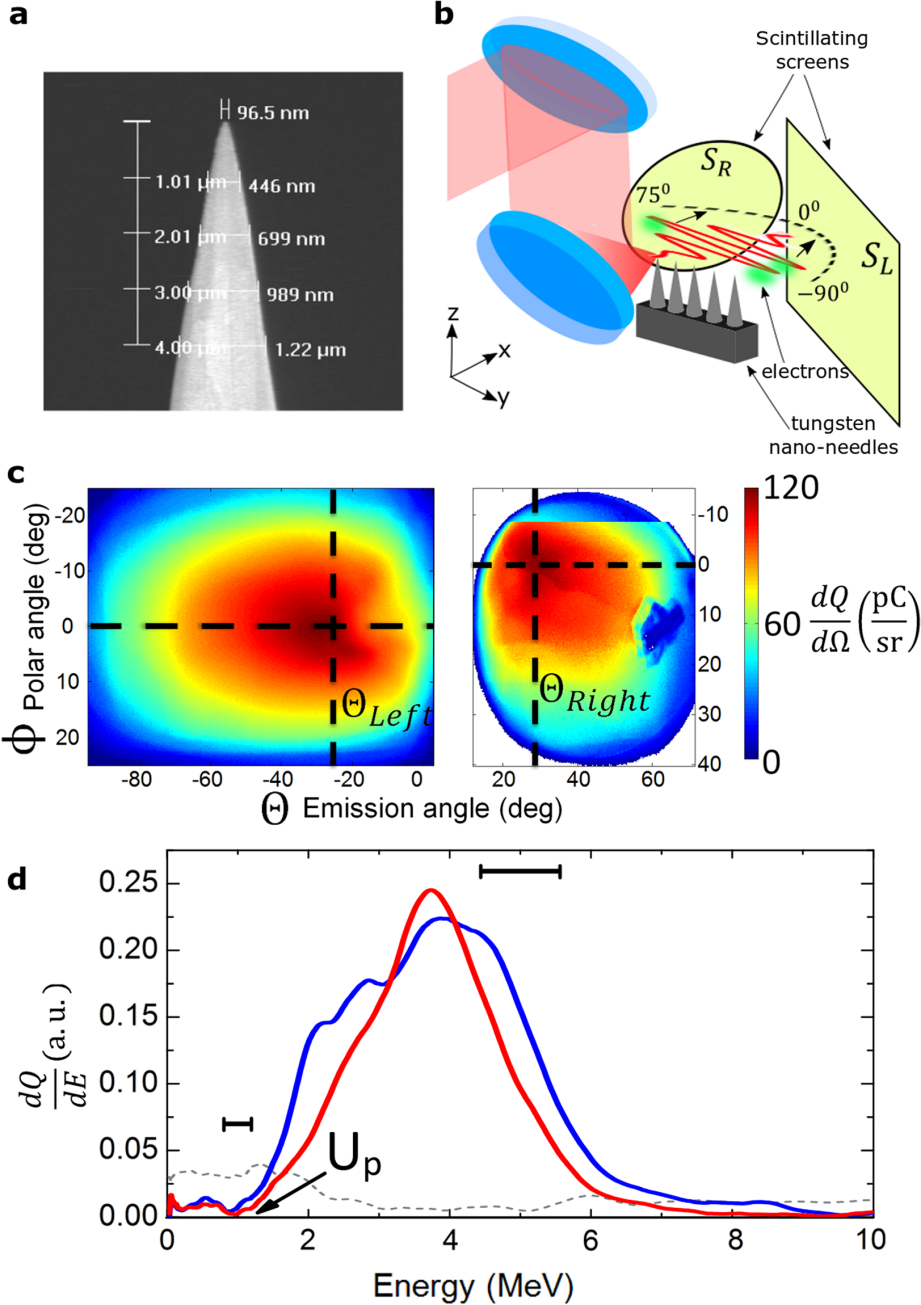
Experimental setup and basic electron beam properties. (**a**) Magnified image of a nano-needle (AFM probes, ©Bruker). (**b**) The laser pulse is tightly focused onto a tungsten nano-needle and the generated electron beams are detected by scintillating screens S_L_ and S_R_. (**c**) Electron angular distribution averaged over 46 shots at of *I*_0_ ≈ 6 × 10^19^ Wcm^−2^ showing two highly divergent electron lobes. The black dashed lines mark the maximum of the emission on both screens. Due to spatial limitation of S_R_ the electron emission angle is referenced to S_L_, peaking on average about ${\theta }_{{\rm{Left}}}\approx -\,25^\circ$. (**d**) Two typical single-shot electron spectra (after subtracting the background, which is plotted with dashed gray line), reaching energies beyond the ponderomotive energy, ${U}_{p}\approx 1.2$ MeV. The two black horizontal bars indicate the resolution at the given electron energies.

In our experiments electron emission was studied by tightly focusing a laser pulse [full-width-at-half-maximum (FWHM) spot diameter ${\sigma }_{{\rm{FWHM}}}=1.22$ µm] with an estimated temporal contrast of 10^17^ from the 16 TW, sub-5 fs Light Wave Synthesizer 20 system (see SM) with *p*-polarization on the tip of a nano-needle (electric field is perpendicular to the needle as shown in Supplementary Fig. S1), which was replaced for each laser shot (see Fig. 1a). Most of the electron emission took place perpendicularly to the needle in the laser polarization plane and was measured by two scintillating screens (see SM), S_L_ and S_R_ (see Fig. 1b), covering an angle of almost ±90° off the laser propagation direction and a third screen S_T_ along the needle axis. Figure 1c shows an average of the angular distributions at an ultra-relativistic peak intensity of *I*_0_ ≈ 6 × 10^19^ Wcm^−2^, consisting of multi-10 pC divergent electron bunches propagating about ${\theta }_{\mathrm{Left},\mathrm{Right}}\approx \pm \,25^\circ$ off the laser axis and Fig. 1d presents peaked electron spectra with careful background subtraction (background is plotted with dashed gray line) extending up to 7–9 MeV measured in 40° with a dipole magnet. It is expected that electrons with even higher energy are propagating in 25° direction. Similarly, divergent lower-charged beams were measured on S_T_, and furthermore, the emission was highly sensitive to polarization as shown in Supplementary Fig. S1.

### Intensity-dominated interaction

**Figure 2:**
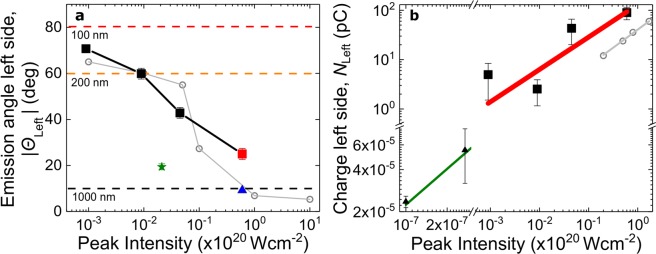
Intensity and target-size dependent electron beams. (**a**) Emission angle of electrons $|{\theta }_{{\rm{Left}}}|$ as a function of intensity, which is controlled by changing the pulse duration, i.e. chirping the laser pulses (full red square: unchirped, full black squares: chirped pulses). Emission angle dependence on target size by aiming at a thicker part of the needle (blue triangle) as well as on target size and intensity by shifting the target out of focus (Z-scan, green star). Simulated emission angles^35^ (open gray circles) by changing the laser energy (see Methods). Dashed lines correspond to Mie angles at the given radii. (**b**) Emitted charge measured on S_L_ (full squares) as a function of the peak laser intensity in the chirp scan. It follows a scaling of ${I}_{L}^{0.66\pm 0.21}$ (fit: red line), similar to previous results using nano-spheres at 1–3 × 10^13^ Wcm^−2^ (full triangles)^4^, i.e. at ${10}^{-6}{I}_{0}$ with a scaling of ${I}_{L}^{0.85}$ (fit: green line). Emitted charge from 3D PIC simulations (open circles), following a similar growth of ${I}_{L}^{0.73}$ (fit: gray line).

The detected electron charge ${N}_{{\rm{Left}}}$ and emission direction ${\theta }_{{\rm{Left}}}$ were studied as a function of target radius *R* and laser intensity *I*_*L*_ by changing the pulse duration. During the scans, the angular divergence of the electron beams remained similar to that in Fig. 1c. $|{\theta }_{{\rm{Left}}}|=|{\theta }_{{\rm{Left}}}|({I}_{L},\,R)$ and ${N}_{{\rm{Left}}}={N}_{{\rm{Left}}}({I}_{L})$ are shown in Fig. 2a,b, respectively. The classical Mie regime is recovered when shooting at ${I}_{L}\approx {10}^{17}$ Wcm^−2^, after chirping the pulses. At lower intensities the emission angle ${\theta }_{{\rm{Left}}}$ shifts towards 90°, reaching a maximum deviation of ≈70°, which matches the theoretical Mie angle of a 100 nm < *R*_eff_ < 200 nm radius target (see SM). The target size was scanned in two different ways: (i) shifting the target out of focus (Z-scan), so that the spatially expanded laser beam interacts with a larger portion of the target (green star in Fig. 2a, where $R\approx 6{R}_{{\rm{tip}}}$) at a lower intensity ${I}_{L}\approx {10}^{18}$ Wcm^−2^ determined by the laser divergence; and (ii) focusing to a thicker part of the needle (blue triangle in Fig. 2a, where $R\approx 10{R}_{{\rm{tip}}}$). In both cases, the interaction with a larger target resulted in a more forward-directed electron emission: $|{\theta }_{{\rm{Left}}}({R}_{1})| > |{\theta }_{{\rm{Left}}}({R}_{2})|$, for ${R}_{1} < {R}_{2}$, regardless of the laser intensity as observed in Fig. 2a. These findings clearly indicate that some characteristic Mie features, i.e. that a larger target results in an electron emission towards the laser propagation direction, are still valid at relativistic intensities^32^ and the target size is still nanometric during the interaction. Furthermore, Fig. 2b shows charges up to 90 pC, which scale with intensity as $N\propto {I}_{L}^{0.66\pm 0.21}$, similar to previous theoretical studies^42^. It also puts our results in perspective with respect to other reported experiments using few-cycle lasers at 6 orders-of-magnitude lower intensity on isolated nanoparticles^4^.

### Waveform-dependent relativistic electron emission

**Figure 3:**
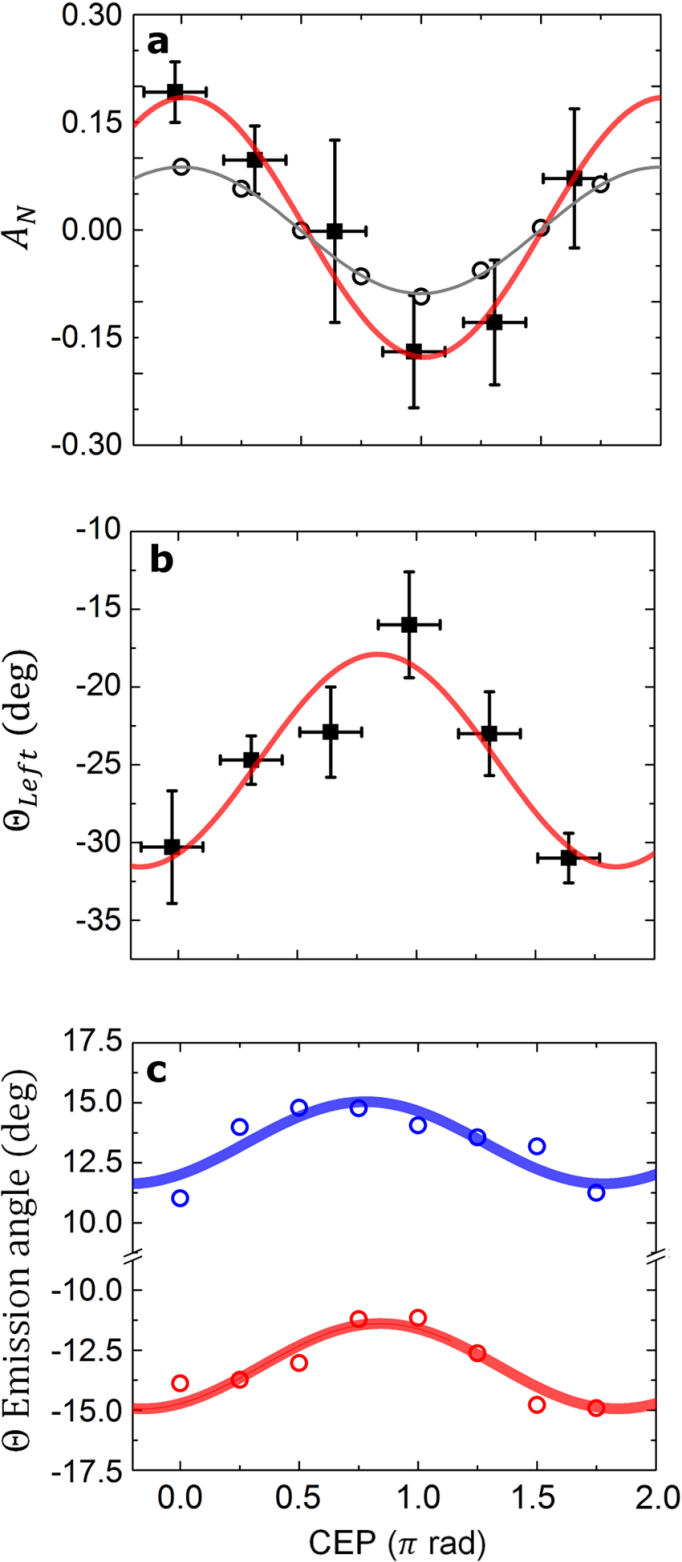
Waveform controlled electron emission. (**a**) Measured (full squares) and simulated (open circles) charge asymmetry parameter with fits as a function of the incoming laser CEP. (**b**) Measured emission angle ${\theta }_{{\rm{Left}}}$ and (**c**) simulated emission angles *θ* with fits as a function of the CEP.

The waveform of the few-cycle driving laser pulses determines the asymmetry between the observables such as *N* or *θ*. The electron yield asymmetry parameter is defined as ${A}_{N}({\phi }_{CEP})=[{\tilde{N}}_{{\rm{Left}}}({\phi }_{CEP})-{N}_{{\rm{Right}}}({\phi }_{CEP})]/[{\tilde{N}}_{{\rm{Left}}}({\phi }_{CEP})+{N}_{{\rm{Right}}}({\phi }_{CEP})]$ (see SM) and Fig. 3a presents the measured ${A}_{N}({\phi }_{CEP})$ with simultaneously observed CEP values for each of the laser shots (46 in total). The electron yield as well as the direction of the electron beam as a function of the CEP oscillated in an anti-correlated (within error bars, see SM) and sinusoidal way in the polarization plane reaching a maximum asymmetry amplitude of 0.18 and 7°, respectively, with a period of 2*π*. These results clearly demonstrate an unprecedented waveform dependence of the entire electron bunch properties in the relativistic interaction, even without any energy filtering.

## Discussion

**Figure 4:**
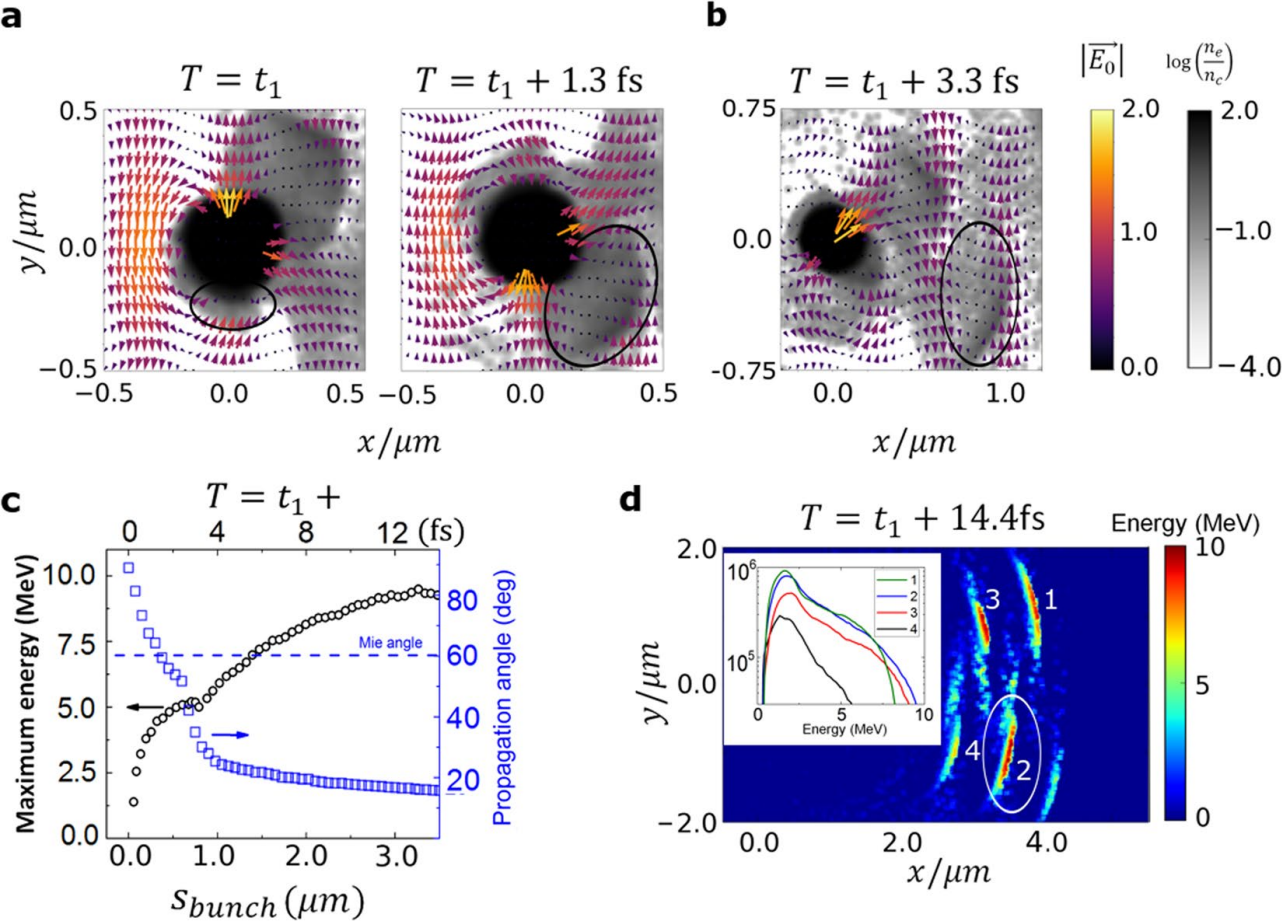
Simulation and analysis of the two-step interaction process. (**a**) First step: Normalized electron density of the nanotarget (grayscale) and normalized electric field (color arrows) during the extraction of an isolated electron bunch (marked with black ellipse) from the target by the near-field at 90° off the laser axis at time *T* = *t*_1_; and its oblique acceleration in the near-field at $T={t}_{1}+1.3\,{\rm{f}}{\rm{s}}$. (**b**) Second step: Normalized electron density (grayscale) and electric field (color arrows) during the vacuum laser acceleration of the tracked isolated bunch at $T={t}_{1}+3.3\,{\rm{f}}{\rm{s}}$. (**c**) Electron energy and propagation angle evolution as a function of the bunch distance to the target surface ${s}_{{\rm{bunch}}}$ and as a function of time. (**d**) Electron energy after approximately one Rayleigh length of propagation at $T={t}_{1}+14.4$ fs. Inset: energy spectra of the individual electron pulses.

3D particle-in-cell (PIC) simulations (see SM) were performed to gain a deeper understanding of the electron emission physics. The overall mechanism relies on a 2-step process, somewhat similar to that in ref.^21^: (1) relativistic electron emission by the enhanced near-field and (2) VLA-type acceleration of these electrons by the high intensity laser pulse in vacuum. Well before the laser pulse reaches the nanotip, the increasing laser intensity turns it into plasma. Although free electrons would quiver with *l*_*q*_ ≈ 1 μm amplitude at the peak of the laser field, the space-charge force of the ions suppresses this oscillation. When the incident laser light couples with the plasmonic field at a later time instant *T* = *t*_1_, the localized enhanced near-field component directed into the target reaches its maximum value of ≈1.3*E*_0_ as depicted in Fig. 4a. This net field pulls a slab of plasma electrons of thickness ${\delta }_{p}^{\ast }$ strongly outwards at about 90° with respect to the laser propagation direction. The charge of the extracted bunch scales^42^ as $N\propto {E}_{L}{\delta }_{p}^{\ast }\propto {I}_{L}^{0.75}$, in agreement with the observations in Fig. 2b. The emission interval is limited to 300 as due to the temporally varying localized SP field and mirrored on the *y* > 0 half-plane after each laser half-cycle^32^. The amplitude of the outward scattering near-field is reduced due to the screening effect of the density gradient from the newly born outward-propagating electron bunch itself.

After surpassing the target’s electrostatic potential (≈3 MeV at ${s}_{{\rm{bunch}}}=100$ nm distance from the target surface), the directly-emitted bunch will not return to the surface^43^ and experiences the near-field (on average 10 TVm^−1^) up to ≈500 nm (a few *l*_*d*_) and gains a maximum energy of 5 MeV. In this region the propagation direction of electrons, plotted in Fig. 4c, is oblique to the laser axis (see Fig. 4a at $T={t}_{1}+1.3\,{\rm{f}}{\rm{s}}$) and resembles that of the near-fields (see Supplementary Fig. S2). Around ${s}_{{\rm{bunch}}}=500$ nm, the first step comes to an end. Hence, the near-field “passes the baton” to the laser for subsequent acceleration in a second phase as indicated for ${s}_{{\rm{bunch}}} > 500$ nm in Fig. 4c. The whole emission is naturally in the sub-half-cycle regime^6^, i.e. the electrons are not rescattered after their emission, because the strong plasmon field (as shown in Supplementary Fig. S3) boosts their energy to relativistic values within a few *l*_*d*_ (see Supplementary Fig. S2).

One optical cycle later, as shown in Fig. 4b, the dense electron bunch is slightly delayed with respect to the laser electric field and sits between two transverse laser field extrema^21,23^. It is then synchronized to and accelerated further by the laser, which has a maximum accelerating field strength of ${E}_{VLA}\approx 3\,{{\rm{T}}{\rm{V}}{\rm{m}}}^{-1}$. The details of vacuum laser acceleration mechanism are beyond the scope of this paper and will be the subject of a future publication. The propagation angle strongly deviates from the original Mie angle (Fig. 2a) mainly due to the forward acceleration by “surfing” on the travelling laser wave^35^, as summarized in Fig. 4c. Although the synchronization prevails well beyond the *Z*_*R*_, i.e. the Rayleigh length, the maximum gained energy of an electron is ${\rm{\Delta }}E\approx 5$ MeV at ${s}_{{\rm{bunch}}}\approx {Z}_{R}$ as shown in Fig. 4d at $T={t}_{1}+14.4$ fs. The final maximum energy is almost 10 MeV, in good agreement with Fig. 1d. Such a high accelerating field ${E}_{VLA}$, is a direct consequence of reaching the VLA regime through a sub-cycle relativistic injection. The magnitude of the overall acceleration field is experimentally supported by the total energy gain and the focusing conditions: ${\rm{\Delta }}E/{Z}_{R}\approx 9\,{\rm{M}}{\rm{e}}{\rm{V}}/4.8\,\mu {\rm{m}}\approx 2$ TVm^−1^. A longer simulation up to 30 μm from the target was performed to confirm the main observations, supporting that the maximum electron energy is unchanged and the attosecond bunch duration is retained even at this propagation distance.

Simulations confirmed that a significant pre-plasma with finite extension was not present in the experiment (see Supplementary Fig. S4). Further PIC simulations at different peak laser intensities support the simple considerations that the first step final energy scales as ${a}_{0}{l}_{d}\propto \sqrt{{a}_{0}}$ whereas the post acceleration step scales with ${a}_{0}$ (see SM), similar to previous studies^29,42,44^. The emission in the first step is intrinsically waveform dependent. As observed in Fig. 3a, about 15–20% of the total emitted charge oscillates periodically. Most importantly, this asymmetry allows the tracked bunch (labeled “2”) to be isolated via energy-filtering. The emitted bunches to left and right do not follow symmetric trajectories, as expected from ponderomotive scattering^27,45^, but the strongest half-cycle bends a bunch (2) the closest to the laser axis in agreement with the experimental observations in Fig. 3b. This bunch has the highest energy, but its charge is lower than the sum of the other two bunches (1, 3) at the other side, which explains the anti-correlation between Fig. 3a,b. An unprecedented degree of control is thus introduced by access to the CEP, which can be used to manipulate the angular distribution.

It should be noted that the acceleration gradients in the present work are an order of magnitude higher than in ref.^21^ thanks to the much tighter focusing and much shorter laser pulse duration which ensures an isolated bunch. Furthermore, using mass-limited nanotargets we have an extra degree of control over the experiment by selecting the size of these targets and thus the charge in electron bunch and indirectly also its energy spread.

In conclusion, we demonstrated relativistic electrons accelerated in the sub-cycle regime from nanoplasmas. Our targets preserve their nanometric scale due to unprecedented temporal structure of the intense pulses. Our experimental findings are consistent with a two-step acceleration process, where the nanoscale near-field and laser vacuum acceleration lead to attosecond MeV electron pulses. This technology provides record-breaking accelerating electric fields strengths beyond TVm^−1^ exceeding all other methods. Our proof-of-principle experiments pave the way to the next generation table-top electron sources and provide new high-energy photon sources. Potential applications by combining ultra-strong few-cycle laser pulses with isolated attosecond relativistic electron bunches could provide a source of energetic X-ray radiation with attosecond pulse durations via Thomson scattering with a counter-propagating laser pulse^46^ very near to the nanotip. Using mass-limited targets the short electron bunch duration can be longer preserved, which possibly allows X-ray generation via free-electron laser seeding or highest temporal and very high spatial resolution via electron diffraction.

## Methods

### Near-field description

The near-field around the nanotarget is described as ${E}_{NF}(r,t)=\alpha {E}_{L}(t)\,\exp \,(\,-\,r/{l}_{d})$, where *α* is the enhancement factor, *l*_*d*_ is the decay length, *r* is the distance from the surface and ${E}_{L}(t)$ is the laser field. The latter is ${E}_{L}(t)={E}_{L,0}(t)\cos ({\omega }_{L}t+{\phi }_{CEP})$, where ${E}_{L,0}(t)$ is the field envelope with a maximum amplitude of ${E}_{L,0}$, ${\omega }_{L}=2\pi c/{\lambda }_{L}$ is the laser angular frequency, *c* is the speed of light, *λ*_*L*_ is the laser wavelength, and ${\phi }_{CEP}$ is the CEP.

### Optical parametric synthesizer

Laser pulses from the 10 Hz repetition rate Light Wave Synthesizer 20 (LWS-20) optical parametric synthesizer system^47^ contain, from 70–75 mJ at the laser, after losses in the beam steering system 40 mJ energy on-target. Its spectrum spans from 580 up to 1020 nm with a central wavelength of 740 nm. It was focused on the tip of a new nano-needle for each shot. The tight focusing using an off-axis parabolic mirror with an f/1 resulted in a FWHM spot diameter of *σ*_FWHM_ = 1.22 μm and a short measured Rayleigh length of *Z*_*R*_ ≈ 4.8 μm, see Supplementary Fig. S5. The compressed pulse duration is reaching sub-5 fs and the corresponding laser peak intensity is ${I}_{0}\approx 6\times {10}^{19}$ Wcm^−2^. The laser is equipped with a CEP phase meter for single-shot CEP tagging^48^. Moreover, nano-scale solid experiments require a very clean laser pulse (excellent high-dynamic-range temporal intensity contrast) of around 7–8 orders of magnitude to avoid early pre-plasma generation that significantly extends the effective target size due to the typical target damage thresholds of ~10^13^ Wcm^−2^ for few-cycle pulses^49^. The LWS-20 has ultrahigh contrast that is estimated to be 10^17^ outside the pump temporal window^50^, i.e. about 30 ps or more before and after the main pulse and 10^7^ at 2 ps. This is sufficient to fulfill the previous requirement, which has been carefully proven not to alter the target at all in the experiments.

### Setup for basic characterization of the relativistic electrons

The electron angular distribution was measured with two absolutely calibrated BIOMAX scintillating screens^51^, S_L_ and S_R_, which were placed a few cm’s away from the target. S_L_ covered an angle range of [−90°:5°] and a smaller circular screen S_R_, [15°:75°] as shown in Fig. 1b. Parameters such as electron directionality ${{\theta }}_{{\rm{Left}}}$ or full electron yield were only measured in S_L_ due to spatial limitations of S_R_. For the sake of completeness, a third screen S_T_ was placed on top of the target covering a solid angle of ≈2.5 sr. The electron spectrum was measured by placing a dipole magnet about 3 cm away from the target after removing S_L_ about 40° off-axis due to spatial limitations in the setup. It had an entrance aperture of 5 × 10 mm^2^ corresponding to about 60 msr solid angle and its exit was covered with scintillating screens which were imaged to a CCD outside the chamber. The influence of X-rays on measured electron charge values by S_L_, S_R_ and S_T_ screens was excluded by comparing the charge obtained in the spectrometer, where the scintillating screen was parallel to the target direction, with the corresponding solid angle on S_L_.

### CEP dependent charge asymmetry parameter

The asymmetry parameter *A*_*N*_ is defined by the integrated electron angular distributions in an equal solid angle to the left and right sides. Thus, integration spans from −75°(75°) to −15°(15°) on S_L_ (S_R_). This corresponds to the whole measured charge on the right side ${N}_{{\rm{Right}}}$, but only a fraction of ${N}_{{\rm{Left}}}$, i.e. ${\tilde{N}}_{{\rm{Left}}}$, due to the reduced solid angle.

### PIC simulations

The 3D PIC simulations were performed with the code EPOCH^52^. The target was a hemisphere with a diameter of 400 nm placed on top of a truncated cone whose upper circular base surface had the same diameter. The density of the target was 100 times overdense (${n}_{e}=100{n}_{c}$). It was irradiated by a Gaussian 4.5 fs (FWHM) laser pulse with polarization perpendicular to the target and a FWHM focal spot diameter of 1.22 µm, reaching a peak intensity of 5 × 10^19^ Wcm^−2^ and *a*_0_ = 4.5 at 740 nm wavelength. The results of Fig. 4, Supplementary Figs S2, S4, 6–7 were obtained with a cosine pulse (${\phi }_{CEP}=0$), while Supplementary Fig. S3 with a sine pulse (${\phi }_{CEP}=-\,\pi /2$). The simulation box was 12 × 12 × 8 µm^−3^ divided in ΔX = 15.63 nm, ΔY = 11.78 nm, ΔZ = 15.63 nm. A full simulation time of 40 fs and a total pseudoparticle number of 2.7 × 10^8^ were used. Further simulations at different peak laser intensities were also performed while typically keeping the other parameters unchanged. A longer simulation, lasting 160 fs and involving a larger box of 12 × 12 × 18 µm^−3^ divided in ΔX = 15.63 nm, ΔY = 11.78 nm, ΔZ = 15.63 nm, was performed to follow the accelerated bunches in vacuum up to 35 µm distance from the target. The scaling of the electron bunch charge with intensity was simulated by varying laser energy instead of pulse duration as in the experiments due to limited computational resources. A comparison of the two intensity control methods was tested with 45 fs pulse duration and 5 × 10^19^ Wcm^−2^ intensity delivering similar angular distribution and charge. Only the CEP triggered asymmetry was not present, see Supplementary Fig. S8.

## Supplementary information

Supplementary Materials

## Data Availability

The data that support the findings of this study are available from the corresponding author upon reasonable request.
