# Supplementary material for: Sub-cycle dynamics in relativistic nanoplasma acceleration
Source: Sci Rep. 2019 May 13;9:7321. doi: 10.1038/s41598-019-43635-3 (PMC6513988; doi:10.1038/s41598-019-43635-3)
Supplement: Supplementary file 1 — Supplementary Materials [file 41598_2019_43635_MOESM1_ESM.pdf]

Supplementary Information to

Title: Sub-cycle dynamics in relativistic nanoplasma acceleration

Authors: D. E. Cardenas[1,2], T. M. Ostermayr[1,2], L. Di Lucchio[3], L. Hofmann[1,2], M. F. Kling[1,2], P. Gibbon[3,4], J. Schreiber[1,2], L. Veisz[1,5]*

Affiliations:

[1]Max-Planck-Institut für Quantenoptik, Hans-Kopfermann Strasse 1, 85748, Garching, Germany.

[2]Ludwig-Maximilian-Universität München, Am Couloumbwall 1, 85748, Garching, Germany.

[3]Forschungszentrum Jülich GmbH, Institute for Advanced Simulation, Jülich Supercomputing Centre, D-52425 Jülich, Germany.

[4]Centre for Mathematical Plasma Astrophysics, Department of Mathematics, KU Leuven, Celestijnenlaan 200B, 3001 Heverlee, Belgium.

[5]Department of Physics, Umeå University, SE-901 87 Umeå, Sweden

*Correspondence to: laszlo.veisz@umu.se

This PDF file includes:

- Supplementary Discussion on the following subjects:
    1. Relativistic effects
    2. Effective target size and density
    3. Determination of the error of CEP
    4. Scaling of the interaction with intensity
- Supplementary Figures: Fig. S1 to Fig. S8
- Supplementary References

Supplementary Discussion

Relativistic effects

In relativistic plasmas, the dielectric constant is given by $\varepsilon_p \approx 1-\frac{\omega_p^2}{\omega_L^2 a_0}=1-\frac{n_e}{n_c a_0}$, where $\omega_p=\sqrt{n_e e^2/(\varepsilon_0 m_e)}$ is the plasma frequency, ε_0 is the vacuum permittivity, n_e is the electron density and $n_c=\omega_L^2 m_e \varepsilon_0/e^2$ is the critical density. Thus, the skin depth is enlarged by relativistic corrections, i.e. $\delta_p=c/\omega_p \rightarrow \delta_p^* \approx c\sqrt{a_0}/\omega_p$. The plasmon decay length of a high intensity-driven nanoplasma in vacuum is determined by $l_d=\frac{c}{\omega_L}\sqrt{\frac{1+\varepsilon_p}{-1}} \approx \frac{\lambda_L}{2\pi}\sqrt{\frac{n_e}{n_c a_0}}$ for a surface plasmon (SP) polariton. As shown in Supplementary Fig. S6c, and d, we observed a similar dependence $l_d \propto a_0^{-1/2}$ in simulations for localized SP with our experimental parameters: $2\pi \mathrm{R}_{eff} \geq \lambda_L$, where R_{eff} is effective target size (see next paragraph). It should be noted that these parameters do not fulfill the condition of the quasi-static regime, which is $2\pi \mathrm{R}_{eff} \ll \lambda_L$. Correspondingly, $l_d/l_q \propto a_0^{-3/2}$ and the sub-cycle emission regime (*1*), which is characterized by $l_d/l_q<1$, is expected for relativistic intensities at a larger extent.

Effective target size and density

The size of a fresh needle is $R_{tip} \approx 50$ nm, see Fig. 1a. However, the effective target size during the interaction is not known due to various reasons such as: (i) a combination of target needle-like geometry and spatial extension of the laser focal spot, i.e. the lower part of the focus hits a thicker portion of the target. This effect alone would lead to $R_{eff} \approx 110$ nm; (ii) small inaccuracy of the transverse needle alignment and laser pointing, which would alone lead to $R_{eff} \approx 90$ nm; and (iii) minor plasma extension before the arrival of the laser pulse. Nevertheless, our results match the theoretical Mie angle of a $100 \text{ nm} < \mathrm{R}_{eff} < 200 \text{ nm}$ radius

target. The estimated degree of ionization of tungsten at our peak intensity is 44 – 45, which corresponds to an electron density of $1400\,n_c$. Using an irradiated volume of 300 nm^3, the electron charge in the interaction volume is about 10 nC, i.e. 100 times more than the observed maximal removed charge. The electrostatic energy to remove another electron beyond the 100 pC is about 10 MeV, which is in agreement with the obtained results.

Determination of the error of CEP

The phase of a focused Gaussian beam shifts $\pm\pi/4$ along the Rayleigh length. The amplitude of this effect is determined by the resolution of our positioning procedure in the longitudinal direction. In this work, each needle was scanned along the laser axis with 1 nm step-accuracy and its position was finally set equidistant to the positions at which the target was out of the focal plane. Thus, the uncertainty is less than the depth of field (DOF) of the microscope (1.8 µm), about DOF/2, yielding an upper limit of CEP uncertainty of approximately $\Delta\varphi_{\mathrm{Gouy}} = \arctan\left(\frac{\mathrm{DOF}/2}{Z_R}\right) \approx 180\ \mathrm{mrad}$. Moreover, the uncertainty from the single-shot CEP meter (*2*) in this experiment was $\Delta\varphi_{\mathrm{CEP\ meter}} \approx 280\ \mathrm{mrad}$. Correspondingly, $\Delta\varphi_{\mathrm{CEP}} = \sqrt{\Delta\varphi^2_{\mathrm{CEP\ meter}} + \Delta\varphi^2_{\mathrm{Gouy}}} \approx 330\ \mathrm{mrad}$.

Scaling of the interaction with intensity

Various extra simulations were performed to compute the dependence of the process on intensities. Supplementary Fig. S6 shows the scaling of relativistic nanophotonics, i.e. the first step. The spectrum of the electron bunch was evaluated and the average electron energy is plotted in Supplementary Fig. S6a as a function of the bunch-target surface distance (s_{bunch}) at three different normalized vector potentials (a_0= 4.5; 8; 20). As the enhanced fields fall exponentially with distance, the energy values are fitted with $E[1-\exp(-s_{\mathrm{bunch}}/l_d)]$ curve,

where E is the electron energy after the first step and l_d is the plasmon decay length including spatial decrease of the field acting on the electron as well as temporal decrease from the enlarging electron phase slippage in the field. Supplementary Fig. S6b indicates the evaluated enhanced electric field at surface (E / l_d) as well as the enhancement factor, i.e. the ratio of this electric field and the laser field in vacuum for the three normalized vector potentials. The enhanced field is certainly linearly increasing with laser field as shown by the fit. Supplementary Fig. S6c illustrates the evaluated decay length from Supplementary Fig. S6a. The fit delivers an approximate $a_0^{-1/2}$ dependence. Supplementary Fig. S6d presents the peak and average (E) electron energies at the end of the first step evaluated from the simulated electron spectra. The fit is in good agreement with the $a_0^{1/2}$ function originating from the relation: electron energy $\propto$ accelerating field $\times$ acceleration length, which is $a_0 l_d \propto \sqrt{a_0}$. Supplementary Fig. S7 shows the scaling of VLA, i.e. the second step, and the full process. On Supplementary Fig. S7a the simulated maximum energy from only the second step is plotted, which is expected to be proportional to the laser field times the acceleration length, i.e. the Rayleigh range. This was confirmed with a long simulation, where after a propagation of $7Z_R$ the maximal energy was the same as after one Z_R. This peak electron energy changes correspondingly linearly with the laser field. Supplementary Fig. S7b illustrates the final maximum electron energy, which scales as the sum of the two steps. It is well visible that the numerical simulations match well the experimental result and the scaling changes approximately at the experimental intensity ($\approx 10^{20}$ Wcm^{-2}). Below this intensity the electron energy gain up to a few l_d in the near-field, i.e. the nanophotonics part, is dominant, while above this threshold the VLA does most of the work on the acceleration process.

Supplementary Figures

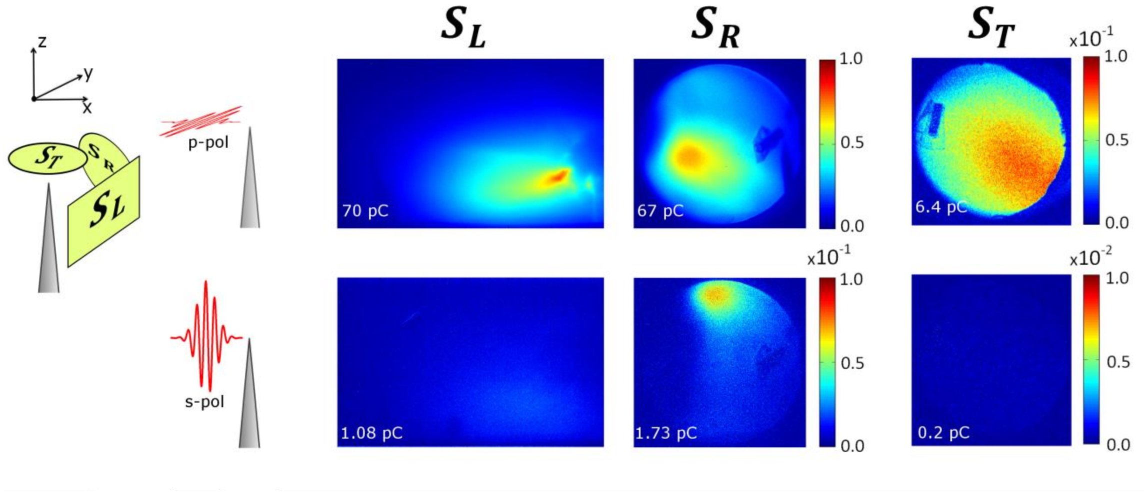

Fig. S1.

Polarization dependent electron emission. Left: geometry with 3 scintillating screens. The laser is focused on the needle and propagates to the right, i.e. x-direction. Right: angular distribution from two selected shots with *p*-polarization, i.e. along y-direction (upper row) and *s*-polarization, i.e. along z-direction (lower row). Color bars are rescaled between the two polarizations. The *p*-polarized laser pulse provides almost 100 times more charge than the *s*-polarized one. Along the needle on S_T the charge is an order of magnitude less in both cases. This is expected as electro-magnetic radiation with only p-polarization generates localized SP at a cylindrical interface in vacuum[3].

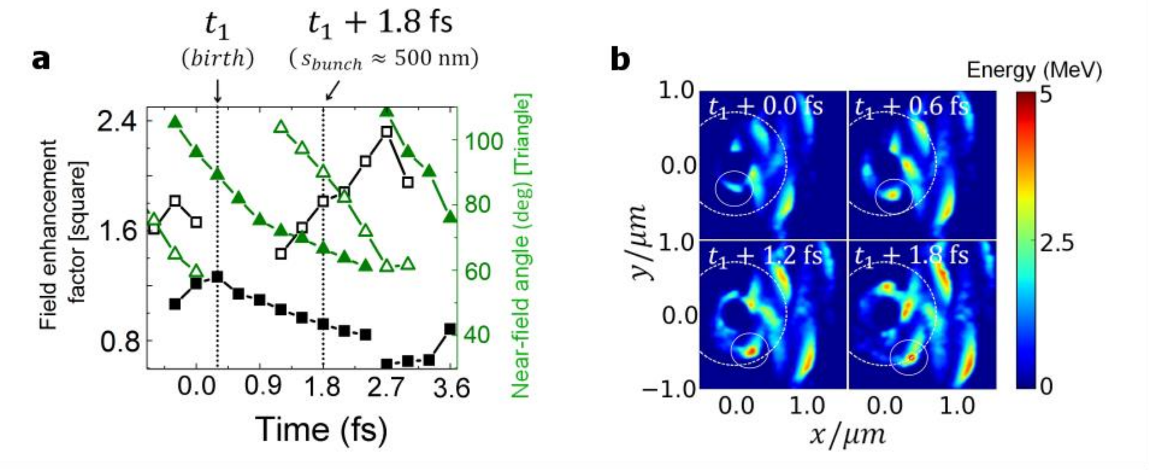

Fig. S2

Simulation of field enhancement and electron energy evolution. **a**, Field enhancement factor (black) and near-field angle (green) for the fields scattering electrons inward (open) and outward the target (full symbols) as a function of time. At the birth (t_1) of the most energetic electron bunch the outward scattering field is maximal, but this maximum is smaller than that of the inward scattering field due to screening by the removed electrons. **b**, Electron energy evolution in real space during the first step of the interaction. The dashed circle corresponding to $s_{\text{bunch}} = 500$ nm around the target indicates the range of the enhanced fields by the nanotarget, i.e. of the first step. The white circle marks the most energetic electron bunch. The sub-cycle regime is visible as the emitted electron bunch just gets rapidly relativistic and does not return to the surface.

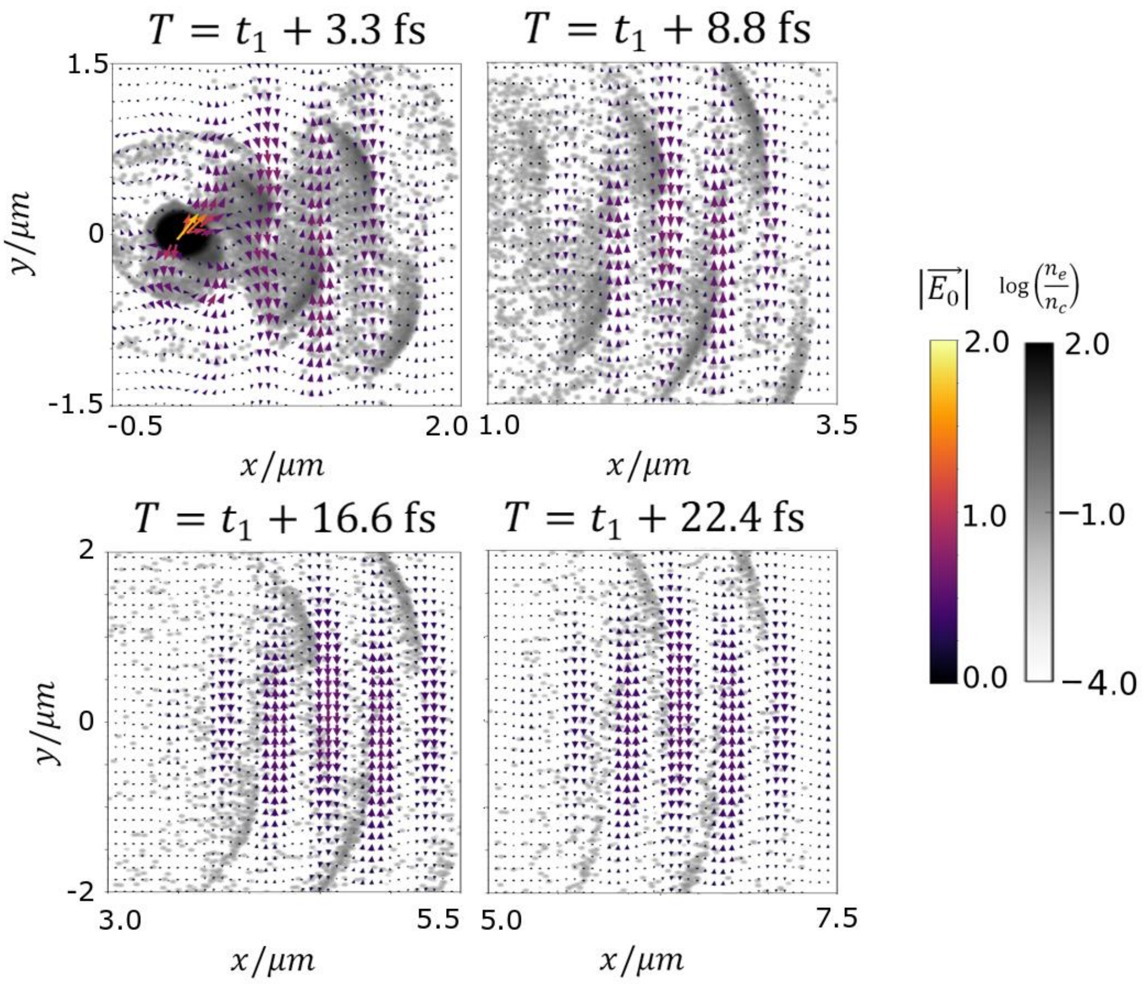

Fig. S3

Sub-cycle regime of relativistic electron emission. Normalized electron density of the nanotarget (grayscale) and transverse y-component of normalized electric field (color arrows) for a sine laser pulse at various times illustrating the sub-half-cycle nature of the process, i.e. the electrons are not rescattered after their emission and propagate between two transverse electric field extrema for a range even beyond the Rayleigh length.

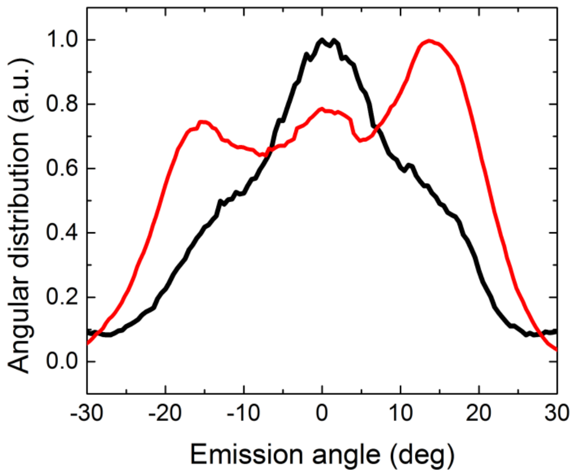

Fig. S4

Simulated influence of plasma expansion. Angular distribution of the electron emission with a steep (red) and an expanded plasma with a scale length of $\lambda/8$ (black) using in both cases the same targets, 4.5 fs (FWHM) laser pulse duration and $5\times10^{19}\,\mathrm{Wcm^{-2}}$ intensity. Non-ideal high dynamic temporal contrast of the laser leads to an early plasma generation and its expansion, which extends the effective target size. The emission is considerably changing from a multiple-peaked (red), where the right and left propagating electrons are separated as in the experiment, to a single broad distribution (black). Also a much weaker CEP dependence is observed with expanded plasma. Therefore, we conclude that this effect is negligible in our experiment.

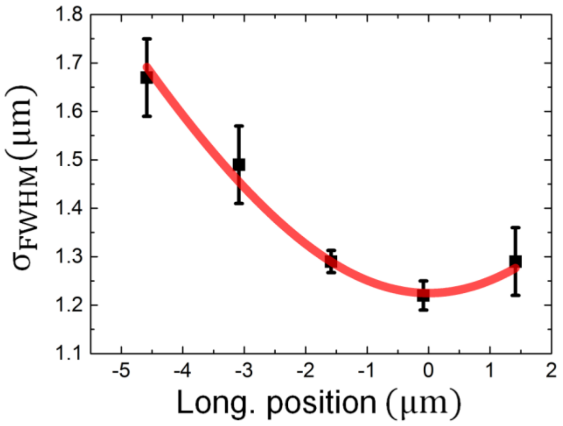

Fig. S5

Measured FWHM laser spot diameter as a function of the longitudinal position. The size in focus is $\sigma_{\mathrm{FWHM}} = 1.22\ \mu\mathrm{m}$ and the Rayleigh range is $Z_R \approx 4.8\ \mu\mathrm{m}$ from a Gaussian beam fit (red line).

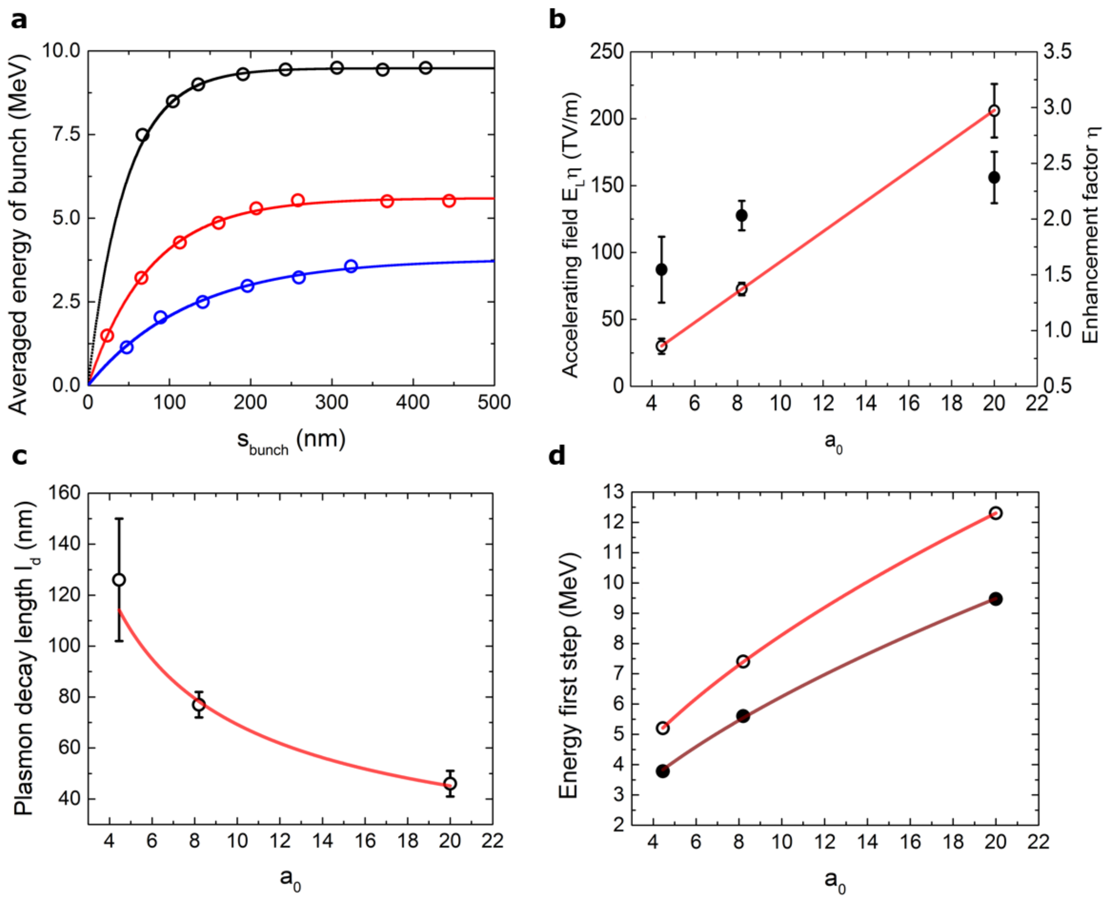

Fig. S6

Simulated scaling of relativistic nanophotonics, i.e. the first step in the interaction. a, Simulated average electron energy as a function of the distance between the bunch and the target surface (s_{bunch}) at three different intensities corresponding to a_0 = 4.5 (blue); 8 (red); 20 (black symbols). The simulated values are fitted with a $E[1-\exp(-s_{\text{bunch}}/l_d)]$ curve (corresponding line). **b**, The evaluated accelerating electric fields at the target surface (s_{bunch} = 0, open symbols) and the associated field enhancement factors (full symbols) as a function of the normalized vector potential. Errors are obtained from fit of the energy vs. s_{bunch} curves plotted in **a**. The accelerating fields are fitted with a linear function (red line). **c**, Evaluated plasmon decay length (l_d) as a function of the normalized vector potential. Errors are obtained from fit of the energy vs. s_{bunch} curve plotted in **a**. The plotted fit provides a $a_0^{-0.62}$

dependence (red line). **d**, Evaluated average energies (E , full symbols) and maximum energies from a different evaluation of the simulated spectra (open symbols) as a function of the normalized vector potential. A fit for average energies delivers $1.55a_0^{0.6}$ and for maximum energies $2.21a_0^{0.57}$ functions (brown and red lines).

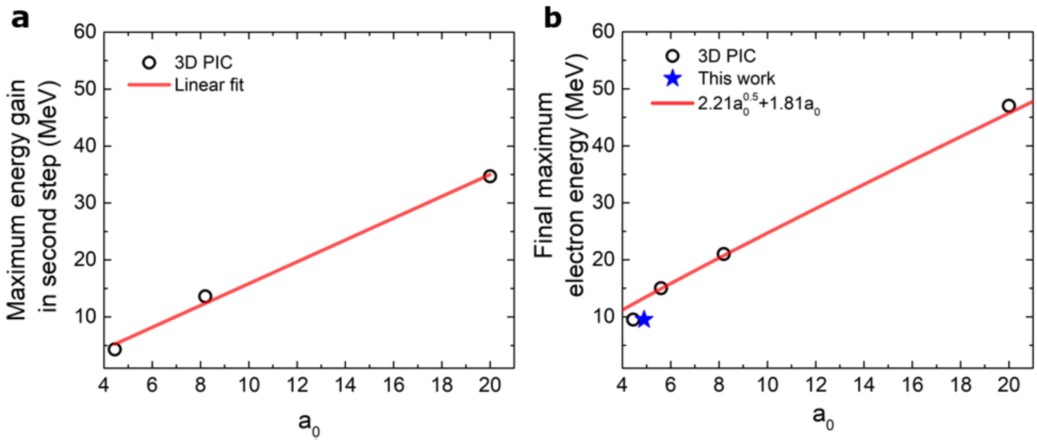

Fig. S7

Simulated scaling of the vacuum laser acceleration, i.e. second step and the full process. **a**, Simulated maximum energy gained only in the second step as a function of the normalized vector potential with a linear fit (red line). **b**, Simulated final maximum energy as a function of the normalized vector potential with $2.21a_0^{1/2}+1.81a_0$ fit (red line). The blue star corresponds to our experimental result.

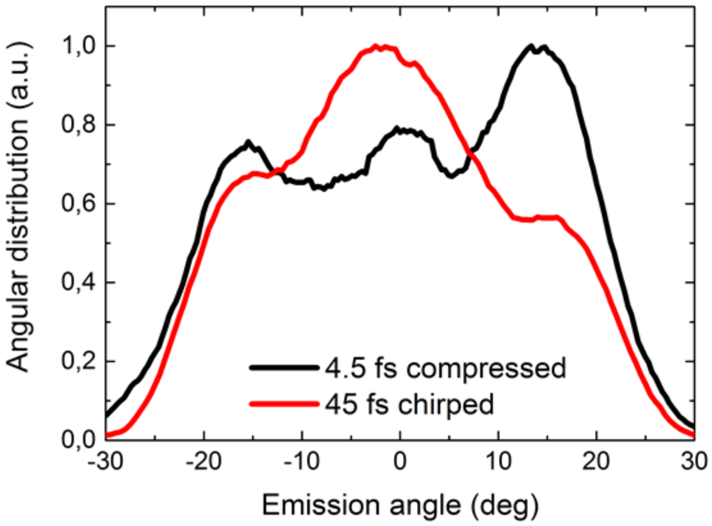

Fig. S8

Simulated angular distribution of the electron emission with different laser pulse duration. 4.5 fs (black) and 45 fs (red) laser pulse duration was simulated with the same intensity of 5×10^{19} Wcm^{-2}. The angular distribution as well as the charge (23.8 pC with 4.5 fs and 21.1 pC with 45 fs) is similar. The only difference is the missing CEP-triggered asymmetry in the case of long pulse. Thus the intensity change in simulations via energy or pulse duration does not significantly influence the results.

Supplementary References:

1. Herink, G., Solli, D. R., Gulde, M. & Ropers, C. Field-driven photoemission from nanostructures quenches the quiver motion. *Nature* **483,** 190–193 (2012).

2. Wittmann, T. et al. Single-shot carrier-envelope phase measurement of few-cycle laser pulses. *Nat. Phys.* **5,** 357-362 (2009).

3. Pitarke, J. M., Silkin, V. M., Chulkov, E. V. & Echenique, P. M. Theory of surface plasmons and surface-plasmon polaritons. *Rep. Prog. Phys.* **70,** 1–87 (2007).
